# Gastroprotective and microbiome-modulating effects of ubiquinol in rats with radiation-induced enteropathy

**DOI:** 10.1186/s42523-024-00320-9

**Published:** 2024-07-19

**Authors:** Walaa A. Eraqi, Walaa A. El-Sabbagh, Ramy K. Aziz, Mostafa S. Elshahed, Noha H. Youssef, Nora M. Elkenawy

**Affiliations:** 1 Department of Microbiology and Immunology, Faculty of Pharmacy, Cairo University, Cairo, 11562 Egypt; 2 Drug Radiation Research Department, National Center of Radiation and Research Technology (NCRRT), Egyptian Atomic Energy Authority (EAEA), Cairo, 11787 Egypt; 3 Microbiology and Immunology Research Program, Children’s Cancer Hospital Egypt 57357, Cairo, 11617 Egypt; 4 Department of Microbiology and Molecular Genetics, Oklahoma State University, Stillwater, OK 74074 USA

**Keywords:** Ubiquinol, CoQ-10, Antioxidant, Gamma radiation, Gut microbiota, Intestinal alkaline phosphatase, Enteritis, Dysbiosis, Albino rats

## Abstract

Radiation enteritis is a frequently encountered issue for patients receiving radiotherapy and has a significant impact on cancer patients' quality of life. The gut microbiota plays a pivotal role in intestinal function, yet the impact of irradiation on gut microorganisms is not fully understood. This study explores the gastroprotective effect and gut microbiome-modulating potential of ubiquinol (Ubq), the reduced form of the powerful antioxidant CoQ-10. For this purpose, male albino rats were randomly assigned to four groups: Control, IRR (acute 7 Gy γ-radiation), Ubq_Post (Ubq for 7 days post-irradiation), and Ubq_Pre/Post (Ubq for 7 days pre and 7 days post-irradiation). The fecal microbiomes of all groups were profiled by 16S rRNA amplicon sequencing followed by bioinformatics and statistical analysis. Histopathological examination of intestinal tissue indicated severe damage in the irradiated group, which was mitigated by ubiquinol with enhanced regeneration, goblet cells, and intestinal alkaline phosphatase expression. Compared to the irradiated group, the Ubq-treated groups had a significant recovery of intestinal interleukin-1β, caspase-3, nitric oxide metabolites, and thio-barbituric reactive substances to near-healthy levels. Ubq_Pre/Post group displayed elevated peroxisome proliferator-activated receptor (PPAR-γ) level, suggesting heightened benefits. Serum insulin reduction in irradiated rats improved post-Ubq treatment, with a possible anti-inflammatory effect on the pancreatic tissue. Fecal microbiota profiling revealed a dysbiosis state with a reduction of bacterial diversity post-irradiation, which was re-modulated in the Ubq treated groups to profiles that are indistinguishable from the control group. These findings underscore Ubq's gastroprotective effects against radiation-induced enteritis and its potential in restoring the gut microbiota’s diversity and balance.

**Supplementary Information:**

The online version contains supplementary material available at 10.1186/s42523-024-00320-9.

## Introduction

Addressing intestinal radiation injury is a major clinical need that is currently largely unmet [1]. Globally, 60–80% of patients receiving pelvic or abdominal radiation therapy experience acute bowel toxicity every year. In the United States alone, 1.6 million patients suffer from post-irradiation intestinal dysfunction annually [2]. Hence, the need for effective intervention is evident. Bowels are among the most radiation-sensitive organs, and those who receive pelvic palliative therapy for colon cancer frequently suffer from radiation enteritis, which typically develops within three months of radiotherapy and is thought to have a detrimental effect on quality of life [2].

Radiation mainly damages the gastrointestinal tract (GIT) through cytocidal, functional, and secondary inflammatory effects. The cytocidal effect of radiation could extend to involve enterocytes, apoptosis, and the microbiome. Radiation also modifies the intercellular environment and releases inflammatory cytokines, ultimately leading to cellular dysfunction [2, 3]. In addition, radiation may induce reversible pancreatitis, which may progress to beta-cells dysfunction, and low insulin levels with increased hyperglycemic and diabetic risk [4]. These events, consequently, increase the incidence of enteric infections and intestinal barrier dysfunction [5].

Markers, such as intestinal alkaline phosphatase (IAP), nitric oxide (NO) metabolites, and cytokines provide valuable insights into the pathophysiology of radiation enteritis. IAP is a member of the alkaline phosphatase enzyme family specifically secreted by enterocytes into the blood circulation and intestinal lumen. It plays a crucial role in the maintenance of gut homeostasis by preserving duodenal pH, promoting mucosal tolerance to the gut microbiome, and detoxifying lipopolysaccharide (LPS) endotoxins that are released from the disintegrated cell walls of dead Gram-negative bacteria [6, 7]. Moreover, it stimulates intestinal bacterial growth via reversal of ATP-induced suppression of normal microbial growth [8]. Dysregulation of IAP is associated with intestinal inflammation and mucosal barrier disruption, including radiation enteritis. [9, 10].

The gut microbiome comprises around 100 trillion microbial cells that reside in the intestinal lumen, where they play an important role in substrate degradation, nutrient absorption, intestinal motility, and immune homeostasis [2, 11]. Radiation enteropathy characterized by symptoms of diarrhea, constipation, malabsorption, abdominal pain, fistula, rectal pain, and potential complications like rectal bleeding or intestinal perforation, is closely linked to dysbiosis, an imbalance in the gut microbiome [11–13]. Dysbiosis not only contributes to enteropathy, but is also closely connected to other medical conditions, including obesity, heart failure, hypertension, Alzheimer’s disease, osteoarthritis, and colorectal cancer diseases [6, 11, 14–18].

Numerous studies, conducted in animal models, demonstrated that exposure to gamma radiation significantly impacts the composition and diversity of the gut microbiome. Despite variations among studies, a notable consistency is observed regarding specific microbial responses to ionizing radiation. The relative abundance of Proteobacteria, Verrucomicrobia, and *Alistipes* was consistently higher post-exposure [19–22], while that of Bacteroidota, Firmicutes, and *Lactobacillus* was consistently lower [21, 23, 24]. Significant reduction in both alpha diversity, assessed by various indexes, such as Shannon, Simpson, ACE, or Chao1, as well as beta diversity, was also observed [24–27].

The gut microbiome maintains luminal epithelial functions via controlling mononuclear phagocyte functions, which eliminates microbes and apoptotic cells. In cases of signal alterations between the microbiome and epithelial cells, the release of interleukin-1β (IL-1β), IL-22, and IL-23 support intestinal barrier repair and anti-microbial defense pathways [28, 29]. NO metabolites are overproduced as part of an inflammatory bowel response, which enhances the depletion of beneficial butyrate-producing microbiota [30]. On the other hand, the gut microbiome activates peroxisome proliferator-activated receptor (PPAR-γ) signals, which have a suppressive effect on nitrate and NF-κB production in addition to their protective effect against oxidation of epithelial luminal cells [31, 32].

Co-enzyme Q-10 (CoQ-10) is an endogenous antioxidant enzyme that is present in all metabolically active tissues and has an essential role in energy homeostasis. It is present in two forms: the oxidized form, ubiquinone, and the reduced form, ubiquinol. Both forms have strong antioxidative capacities, but ubiquinol is more absorbable and bioavailable than ubiquinone owing to the hydrophobic property of ubiquinone. It protects DNA, proteins, and lipids from oxidation, in addition to guarding the cell membrane maintaining endothelial functions [33, 34]. Although ubiquinol has a powerful free radical-scavenging activity and protects normal tissues from radiation’s damaging effect, it intriguingly does not interfere with radiation’s cytotoxic effect on tumors either in vitro or in vivo [35, 36].

Previous studies examining the effects of CoQ-10 on microbiomes have shown notable shifts in microbial composition and function post-supplementation. Findings have indicated that CoQ-10 plays an important role in reducing dysbiosis, increasing both alpha and beta microbial diversity, and improving the intestinal integrity, indicating better gut health and metabolic function. CoQ-10 alters the composition of the gut microbiota, favoring the proliferation of beneficial bacteria, like *Lactobacillus* and *Ruminococcus*, while reducing potentially harmful species [37, 38]. It is hypothesized that the antioxidant properties of CoQ-10 contribute to these effects by reshaping the gut microbiota [38]. Thus, we aimed to investigate the possible role of ubiquinol (more bioavailable form of CoQ10), well known for its antioxidant properties, in the modulation of gut microbiome in rats subjected to gamma radiation-induced enteropathy and its possible effect on recovering the intestinal homeostasis affected by radiation.

## Material and methods

### Animals

Male albino rats, weighing 200–230 g., were obtained from the animal house of the National Research Center, Giza, Egypt. Animals were acclimatized for one week before the beginning of the experiment and given a standard pellet diet and water *ad* libitum in a controlled environment at temperature of 25 ± 2 °C, constant relative humidity, and a 12-h dark/light cycle.

### Ethical statement

This work was conducted according to the principles of the “Guide for the Care and Use of Laboratory Animals” published by the US National Institutes of Health (NIH Publication No. 85-23, revised 2011) and approved by the Research Ethics Committee (REC) of the Faculty of Pharmacy, Cairo University (approval # MI3236).

### Drug

Ubiquinol, the reduced form of Ubiquinone (Co-Q10), was obtained from CanPrev® (Premium Natural Health Products, Canada) in the form of 100 mg soft gel capsules. It was dissolved in 1% tween 80 solution and administered to rats in a dose of 10 mg/kg, p.o. (*per os*, i.e., oral) daily, according to Kiremitli et al. [39].

### Irradiation of animals

The whole body of rats was exposed to a single acute dose of γ-radiation, 7 Gy, according to Kakleri et al. [1] and Trajkovic et al. [40]. The animals were irradiated with a Gamma Cell-40 biological irradiator (137Cs irradiator unit, Sheridan science and technology park, Mississauga, Ontario, Canada). The dose rate was 0.33 Gy/min at the time of the experiment. All animals were irradiated at the same time of day (12:00 ± 1 h) to avoid circadian variation and radiosensitivity.

### Experimental design

Male rats (n = 32) were used in this experiment to avoid the effects of female hormones. The treatment schedule was determined based on pilot histomorphometry examination, which confirmed significant intestinal damage after 7 days as compared to 14 days post-irradiation (Supplementary Fig. S1). After acclimatization, rats were randomly allocated into four groups (n = 8); the first group was not irradiated and received the vehicle daily (1% tween 80) (Control); the second group was irradiated at a dose of 7 Gy (IRR) and received only daily dose of the vehicle; the third and fourth groups were also irradiated as described above and treated daily with Ubq for either 7 days following irradiation (Ubq_Post), or 7 days before irradiation and another 7 days following irradiation to reach 14 days of treatment (Ubq_Pre/Post).

On the 15th day of the experiment and after a prior night fast, rats were anesthetized with intraperitoneal ketamine (80 mg/kg). Blood samples were collected via cardiac puncture and centrifuged to obtain serum samples and then death was ensured via decapitation. Rats were dissected to obtain intestinal (jejunum) samples. These samples were subsequently washed and further subdivided into two parts: one part was homogenized in PBS and stored at − 80 °C for biochemical investigation, and the other part was fixed in 10% formalin for histopathological examination.

### Intestinal histomorphometric analysis

Intestinal tissue samples were fixed in 10% neutral buffered formalin solution for histopathology. Tissue specimens were processed as follows: dehydrated in an ascending concentration of ethanol, cleared in xylene, embedded in paraffin wax, and sectioned at 5-micron thickness. Prepared slide sections were stained by hematoxylin, eosin, and periodic acid Schiff for the detection and counting of goblet cells [41].

Intestinal lesion scores of 0 (none), 1 (mild), 2 (moderate), and 3 (severe) were assigned according to the following semi-quantitative scale: (1) surface and crypt epithelium degeneration, (2) villus structure degeneration, and (3) inflammatory cell infiltration. For each rat, the microscopic score was calculated as the sum of the scores given to each criterion in five different pathological sections, in which at least five microscopic areas were examined per specimen. A score of nine expressed the most severe damage to the small intestine [42].

### Immunohistochemical investigation of IAP

Rats’ intestine sections were processed for staining with IAP (IgG) antibody according to manufacturer instructions (GeneTex ® Co, California, USA, Catalog No. GTX112100). Expression of IAP was detected with the brown color expression under light microscope.

### ELISA assays of IL-1β, Caspase-3, PPAR-γ and serum insulin

Intestinal homogenate was used for assessment of IL-1β, caspase-3 and PPAR-γ using Rat IL-1β (Elabscience®, catalog no. E-EL-R0012, USA) Rat Caspase-3 (CusaBio®, catalog no. CSB-E07264r, USA) and Rat specific PPAR-γ (Elabscience®, catalog no. E-EL-R01184, USA) ELISA kits, respectively, according to the manufacturer instructions.

Rat-specific insulin ELISA kit was used for measuring insulin in serum samples following the manufacturer guidelines (Cloud-Clone Corp®, catalog no. CEA682Ra, USA). Tukey–Kramer multiple comparison’s test was selected to carry out all statistical tests.

### TBARs and NO metabolites assays

An intestinal homogenate was used for UV spectrophotometric analysis of thio-barbituric reactive substances (TBARs) and NO metabolites. TBARs, a lipid peroxidation byproduct, was measured according to Buege and Aust’s method [43]. Briefly, the intestinal tissue homogenate was added to trichloroacetic acid buffer; this acidic medium permit TBARs to produce a pink absorbed color at 535 nm upon reaction with thio-barbituric acid reagent, after boiling, cooling, and centrifugation. The NO metabolites were determined calorimetrically according to Takada et al. [44], based on Griess reaction inhibition, in which dinitrogen trioxide (N_2_O_3_) produced from the nitrous acid formation from nitrite (or autoxidation of NO) in acidic medium reacts with sulfanilamide to produce a diazonium ion which is then reacted with *N*-(1-napthyl) ethylenediamine to form an azo colored product that absorbed strongly at 540 nm. The assay data were tested for significance by ANOVA followed by Tukey–Kramer multiple comparisons.

### Fecal sample collection

Fecal samples were collected at the end of the experiment (seven days after radiation). Each rat was transferred from its home cage to a clean, empty, sterile cage and allowed to defecate naturally. Two fecal pellets were collected for each rat with autoclaved sterile forceps and stored in sterile Eppendorf tube at − 80 °C until DNA extraction.

### DNA extraction

DNA was extracted from the frozen fecal pellets by the QIAamp DNA Stool Mini Kit (Qiagen, Germany), according to the manufacturer’s protocol. DNA was quantified in a Qubit® fluorometer (Life technologies, Carlsbad, CA, USA). The size and integrity of the extracted DNA were evaluated by electrophoresis in an agarose gel (0.5% wt/vol) and visualized with ethidium bromide staining under UV light. DNA was then kept at − 20 °C until further use.

### PCR amplification and illumina sequencing of 16S rRNA genes

The prokaryotic-specific primer pair 515-F and 805-R were used, with modification to include sequencing adapters, to amplify the V4 hypervariable region of the 16S rRNA gene [45]. Gel electrophoresis was used to confirm the amplification process and ensure the lack of detectable contamination. PureLink™ PCR purification kit (Invitrogen, Waltham, MA, USA) and Nextera XT V2 Index kit (Illumina, Inc., San Diego, CA, USA) were used for amplicon purification and barcoding, respectively. Products were then sequenced by the pair-end technology in an Illumina iSeq-100 platform, as previously described [46, 47].

### Processing and analysis of sequencing data

Raw sequences generated from the Illumina iSeq platform were processed and analyzed by the Quantitative Insights Into Microbial Ecology 2 (QIIME2) pipeline [48]. Raw reads were analyzed and assigned to amplicon sequencing variants (ASVs). For preprocessing of sequences, the DADA2 plugin was used in trimming and filtering out noisy sequences (median Phred quality ≥ 25, maximum of two expected errors per read = 2), removing chimeric sequences, merging paired reads, and finally generating a feature table of representative high-resolution ASVs. Taxonomic assignment of the results was performed by a pre-trained Naive Bayesian Classifier against the SILVA reference database (Release 138), at 99% sequence similarity and with a q2-feature-classifier plugin [49].

Afterwards, QIIME2 scripts were used for microbial diversity analysis on the basis of both inter and intra-community features. Rarefaction plots were generated to ensure that each sample was sufficiently sequenced to represent its diversity, and to set an even sampling depth needed in downstream analyses. Alpha diversity was estimated by metrics for richness of communities (Observed Features) and evenness (Pielou’s Evenness), as well as the inclusive Shannon’s diversity index. Beta diversity between the different experimental groups was estimated by the Bray–Curtis measure of dissimilarity, and the results were visualized by three-dimensional principal coordinates analysis (PCoA) with the Emperor tool.

### Data availability

Raw data of 16S rRNA reads were deposited in the NCBI Sequence Read Archive under Accession Number: Bioproject PRJNA1018889 and biosamples (SAMN37454106–SAMN37454120).

## Results

### Intestinal histomorphometric and IAP expression analysis

**Fig. 1:**
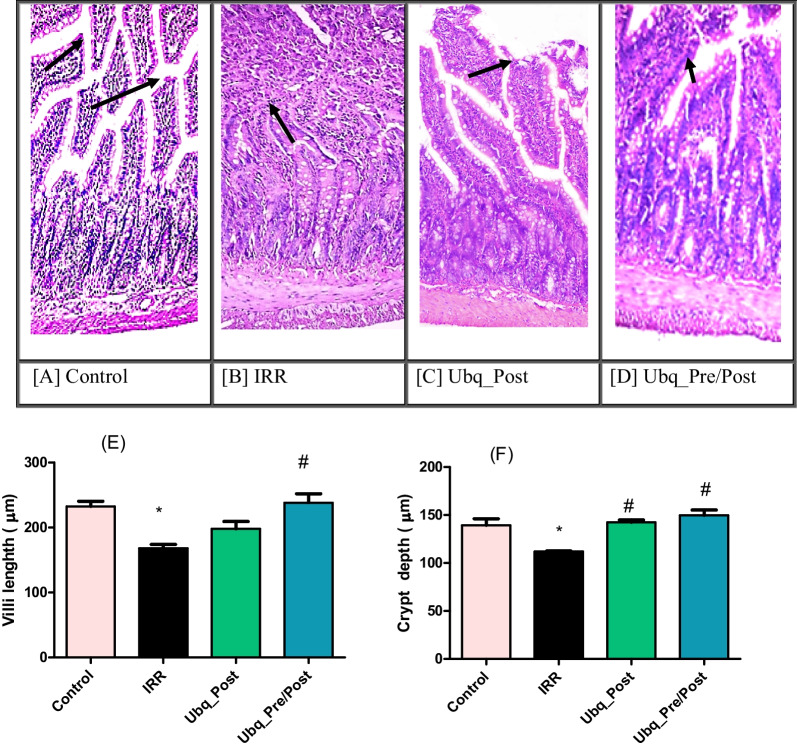
Effects of Ubq treatment (10 mg/kg/day, p.o) on intestinal histomorphometry in irradiated rats (7 Gy): Photomicrographs of intestinal mucosa tissue sections showing **A** normal histological structure of intestinal mucosa in the Control group, **B** severe damage of intestinal villi with loss of goblet cells in the IRR group, **C** mild epithelial shedding at the apices of some intestinal villi with marked loss of goblet cells in the Ubq_Post group, and **D** mild epithelial shedding at the apices of some intestinal villi in the Ubq_Pre/Post group. Bar plots comparing the intestinal villi length in µm (**E**), and the crypt depth in µm (**F**). Data are expressed as mean ± standard error of the mean (S.E.M). Statistical significance was tested by one-way ANOVA followed by Tukey’s Multiple Comparisons test, and *p* ≤ 0.05 was considered significant. *Significant difference against the Control group and #significant difference against the IRR group. The graph was generated by GraphPad Prism (version 5)

**Fig. 2:**
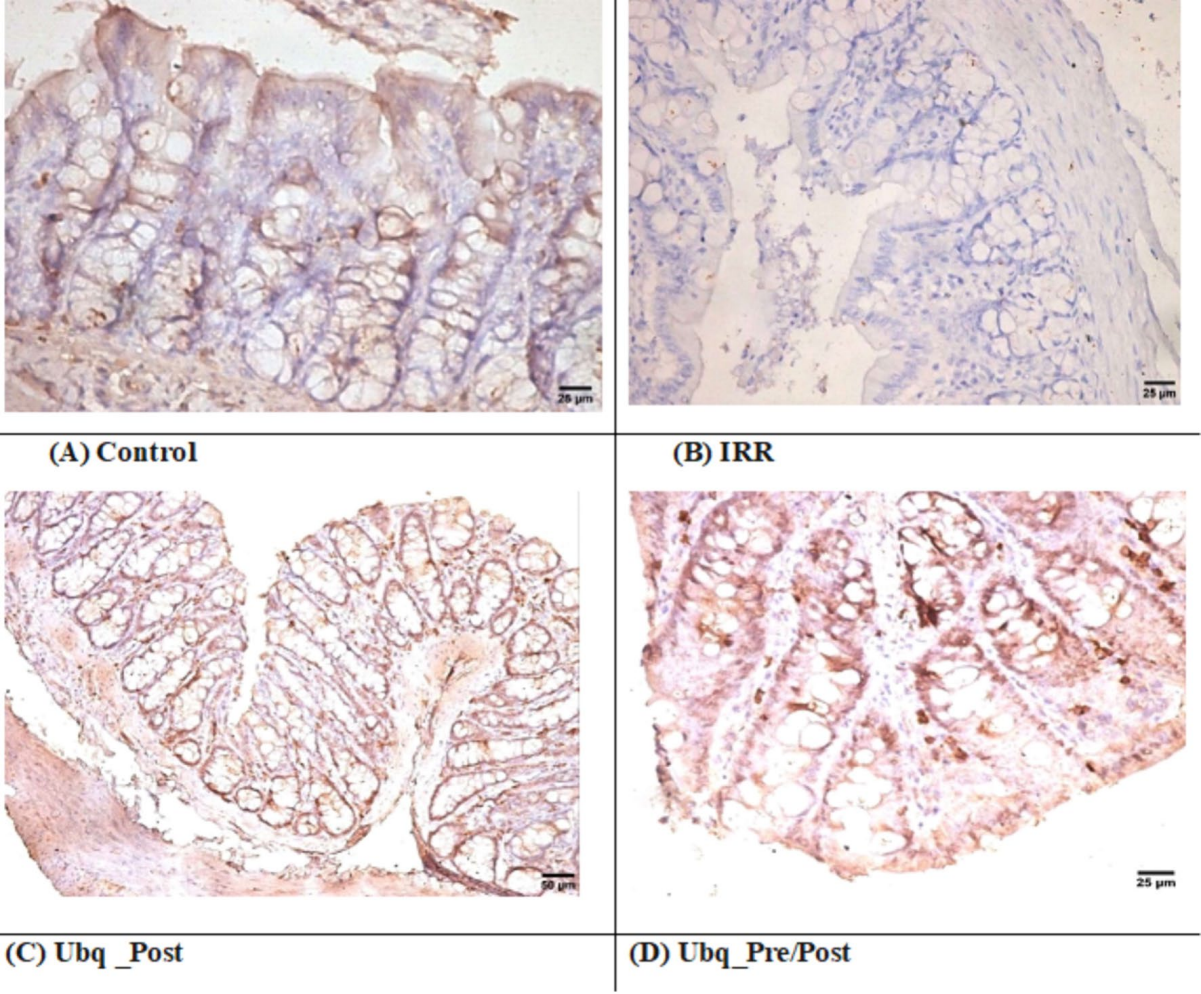
Effects of Ubq treatment (10 mg/kg/day, p.o) on the expression of IAP in the intestinal epithelium of γ-irradiated rats (7 Gy). **A** Positive expression (++) in the intestinal epithelium membrane of the Control group; **B** No detectable expression the in IRR group; **C** and **D** strong expression of IAP (+++) in both treated groups (Ubq_Post and Ubq_Pre/Post)

First, we set out to determine the major phenotypic alterations that gamma irradiation induces in the intestinal tissue and the possible protection by Ubq treatment on the jejunum, which was more negatively affected by radiation than the colon (Supplementary Fig. S2). The intestinal mucosa of the control group had a normal histological structure, and the villi were lined by a single layer of tall columnar cells with oval basal nuclei. Numerous goblet cells were scattered between the columnar cells (score = 0). The intestinal crypts were regularly arranged and lined by columnar epithelial cells (score = 0). The intestinal glands appeared intact and were lined by high cuboidal epithelial cells without any significant pathological alterations (Table 1, Fig. 1A). Significant positive expression of IAP was visualized by IHC- Peroxidase-DAB staining (Fig. 2A).

**Table 1:** Histopathological examination scores in rat small intestine (jejunum)

Histopathological parameters	Control	IRR	Ubq_Post	Ubq_Pre/Post
Degeneration of crypt	0	2	0	0
Degeneration of villus structure	0	3	1	1
Inflammatory cell infiltration	0	2	1	0
Total score	0	7	2	1

On the other hand, the villi of irradiated animals (IRR) were severely damaged and were characterized by desquamation of epithelial lining, with loss of goblet cells (score = 3). Both mucosa and submucosa were infiltrated with lymphocytes and macrophages, with fusion of intestinal villi and shedding of crypt epithelial lining with decrement of intestinal villi length, in addition to hyperplasia of intestinal glands (score = 2, Table 1, Fig. 1B, E, F). IAP visualization in the IRR group reflected its negative expression in the epithelial cells of the intestinal membrane (Fig. 2B).

Animal tissues from the Ubq_Post group had a moderate improvement over those from the IRR group, as the apices of some intestinal villi shed epithelial cells with loss of goblet cells (score = 1), while intestinal crypts had normal lining without significant pathological damage (score = 0). Inflammatory cell infiltration was detected in both mucosa and submucosa (score = 2), while intestinal glands appeared intact with no pathological alterations (Table 1, Fig. 1C).

In comparison, a marked improvement of the intestinal mucosa, in the Ubq_Pre/Post group, appeared as mild epithelial shedding at the apices of some intestinal villi (score = 1) with normal lining of intestinal crypts (score = 0), and maintenance of villi and crypt length (Fig. 1E, F). Numerous goblet cells were scattered along the intestinal villi without detectable inflammatory cell infiltration in neither the mucosa nor submucosa (score = 0). Intestinal glands had intact epithelial lining, with basal basophilic nuclei (Table 1, Fig. 1D). However, in both groups that received Ubq, IAP was highly detected (Fig. 2C, D).

### Effect of Ubq on intestinal IL-1β, caspase-3, TBARs, and NO metabolites

**Fig. 3:**
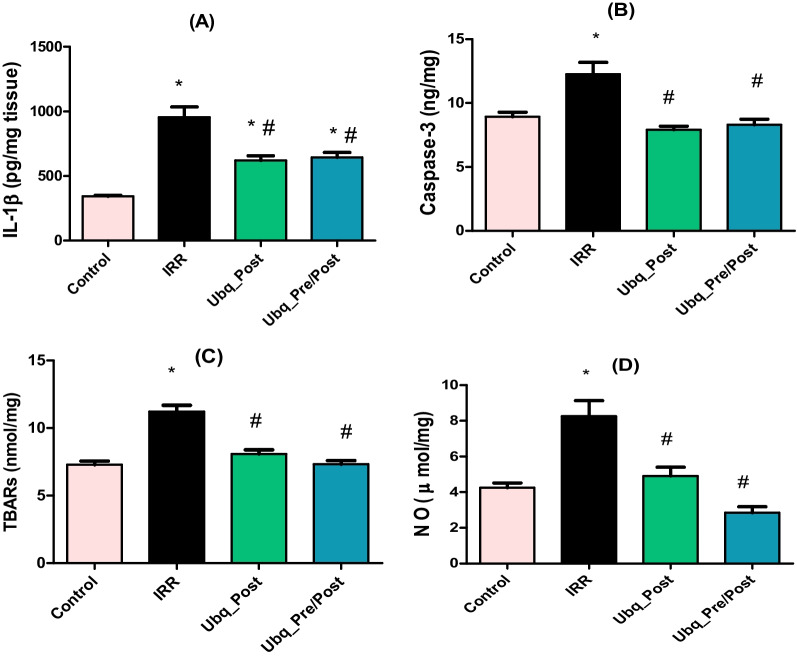
Effects of Ubq treatment (10 mg/kg/day, p.o.) on **A** intestinal IL-1β (pg/mg), **B** intestinal caspase-3 activity (ng/mg) **C** intestinal TBARs (nmol/mg), **D** intestinal NO (μmol/mg). IL-1β and caspase-3 were quantified by ELISA, while TBARs and NO were determined spectrophotometrically. Data are expressed as mean ± S.E.M. Statistical significance was tested by one-way ANOVA followed by Tukeyʾs Multiple Comparisons test, and *p* ≤ 0.05 was considered significant. *Significant difference against the Control group and #significant difference against the IRR group. The graph was generated by GraphPad Prism (version 5)

Irradiation of rats with an acute dose of 7 Gy induced a significant increase in IL-1β, the key modulator of intestinal inflammation, by 178.15% compared with normal values in the control group. Treatment with Ubq for 7 days after irradiation induced a significant decrease in intestinal IL-1β by 35.08%, compared with irradiated non-treated rats. The same result was attained upon treatment with Ubq for 14 days, i.e., 7 days before and 7 days after irradiation (Fig. 3A).

Gamma radiation-induced oxidative stress markers, NO metabolites and TBARs, were evaluated in intestinal homogenates of all groups to assess the generation of reactive oxygen species (ROS). Irradiation induced significant increments in NO and TBARs levels by 94.48% and 54.2%, respectively (Fig. 3C, D). Treatment with Ubq either only after irradiation (Ubq-Post) or before and after irradiation (Ubq_Pre/Post), significantly decreased the levels of NO and TBARs, without significant changes between the two treated groups (Fig. 3C, D).

Caspase-3 was measured in this study as a marker of the ability of Ubq treatment to control irradiation apoptosis in intestinal cells. Ubq treatment, for either 7 or 14 days, significantly reduced intestinal caspase-3 activity in irradiated rats by 35.31% and 32.25%, respectively (*p* ≤ 0.05). However, no significant difference between the caspase-3 activity of the Ubq_Post and Ubq_Pre/Post groups was observed (Fig. 3B).

### Effect of Ubq on intestinal PPAR-γ and serum insulin level

**Fig. 4:**
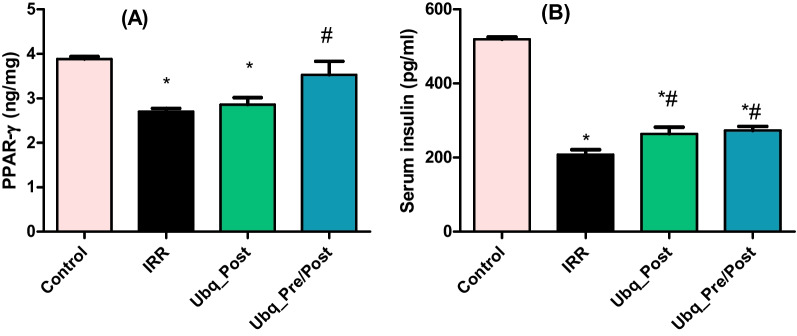
Effects of Ubq treatment (10 mg/kg/day, p.o.) on **A** intestinal PPAR-γ (ng/mg) and **B** serum insulin level (pg/ml), both determined by ELISA. Data are expressed as mean ± S.E.M. Statistical significance was tested by one-way ANOVA followed by Tukey’s Multiple Comparisons test, and *p* ≤ 0.05 was considered significant. *Significant difference against the Control group and #significant difference against the IRR group. The graph was generated by the GraphPad Prism software (version 5)

Significant decrements in intestinal PPAR-γ and serum insulin were observed after 7 days of acute alter to γ-radiation (7 Gy) by 30.49% and 59.91%, respectively (Fig. 4A, B). Irradiated rats in the Ubq_Pre/Post group exhibited normalization of intestinal PPAR-γ (Fig. 4A). Although insulin levels in the Ubq_Post and Ubq_Pre/Post groups significantly increased (26.9% and 31.27%, respectively) compared with irradiated non-treated values, insulin levels in both groups were still significantly lower than those of the control group (Fig. 4B).

### Ubq restores gut microbiota diversity in gamma-irradiated rats.

After iSeq sequencing and data preprocessing, 101,515 high-quality sequence reads were included in downstream analyses.

#### Alpha diversity patterns

**Fig. 5:**
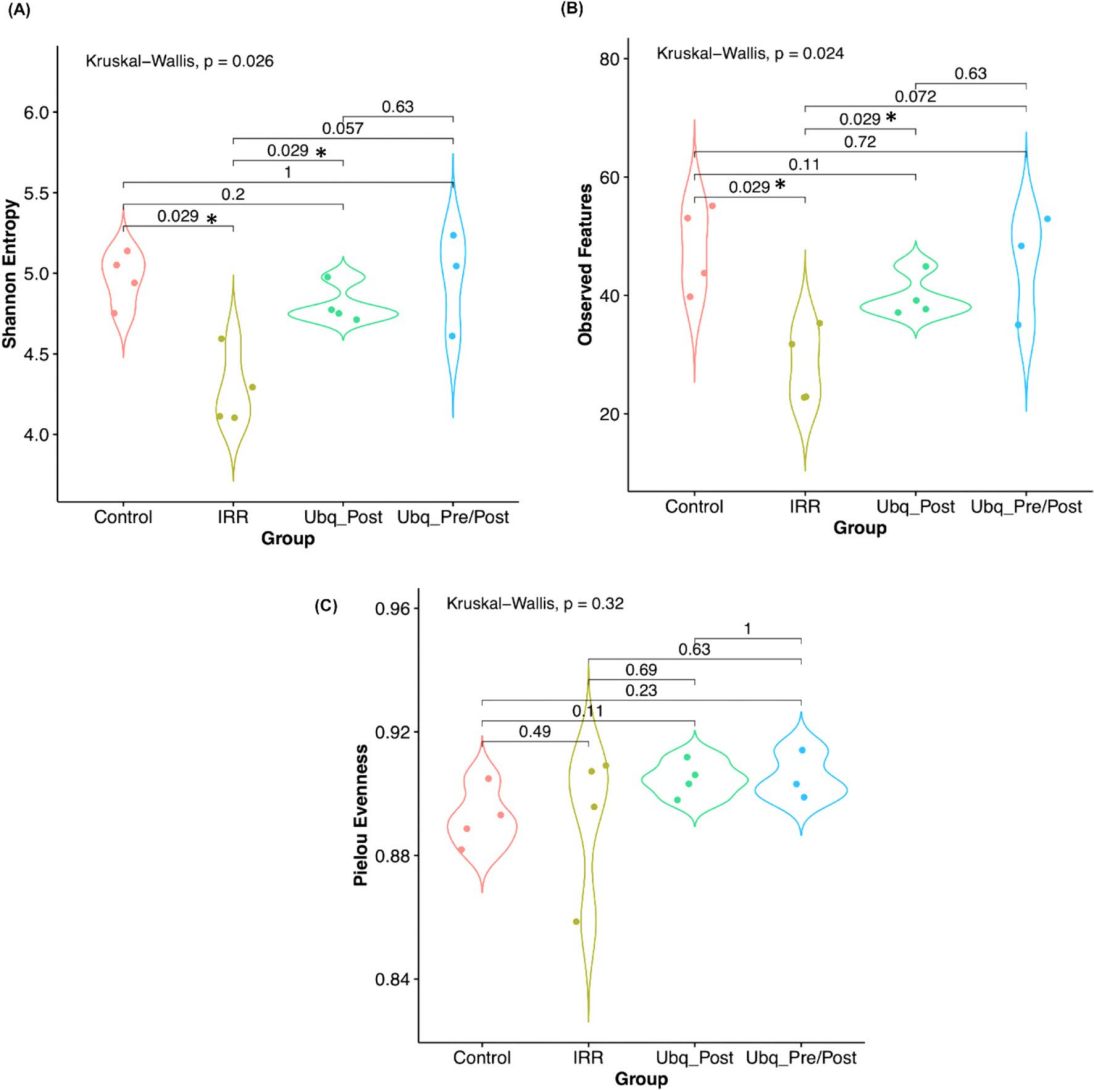
Bean plots representing the gut microbiome alpha diversity, expressed by the following indices: **A** Shannon’s diversity index **B** Number of observed species, and **C** Pielou’s Evenness. The tested groups are plotted on the X-axis while alpha indices were plotted on the Y-axis. Statistical significance of pairwise comparisons was calculated by the Kruskal–Wallis test, followed by post-hoc pairwise Dunn’s test. Asterisks (*) indicate *p* value ≤ 0.05

Alpha diversity analysis, expressed as richness (observed features) or Shannon’s diversity index, demonstrated that irradiation induced a significant decrease in diversity in the IRR group microbiota, in comparison to that of untreated (Control) rats (Kruskal–Wallis test, *p* < 0.05). Furthermore, the gut microbiomes of both Ubq_Post and Ubp_Pre/Post groups were significantly more diverse than those of the gamma-irradiated group (pairwise Wilcoxon test, *p* = 0.04, *p* = 0.02, respectively), and with no significant difference from the randomized control group (Fig. 5A, B). This diversity difference, however, was not reflected in feature evenness, as the Pielou’s evenness index was not significantly different between the four tested groups (Kruskal–Wallis *p* = 0.32, Fig. 5C). Rarefaction curves of diversity estimators reached plateau phase for all samples (Supplementary Fig. S3), indicating that most of the bacterial community had been captured. Collectively, these results clearly demonstrate that Ubq administration before and after—or even only after—irradiation restores the alpha diversity of the gut microbiome of irradiated mice to levels comparable to those not receiving radiation.

#### Community structure patterns

**Fig. 6:**
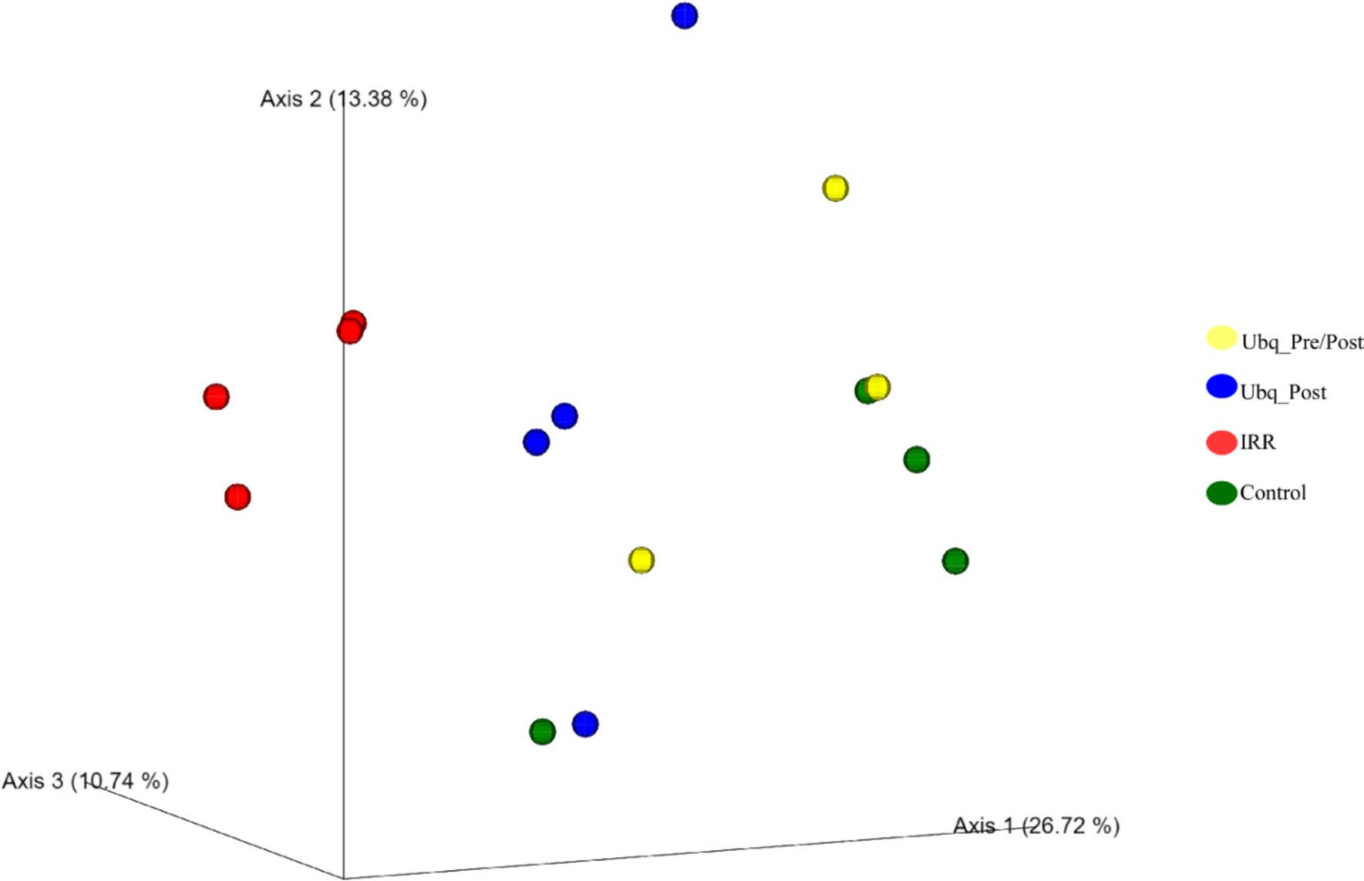
Principal coordinate analysis (PCoA) of gut microbiome composition based on Bray–Curtis measure of dissimilarity. The percentages of the community variation explained are depicted

Ordination plots constructed with Bray–Curtis dissimilarity index and visualized by principal coordinate analysis (PCoA) demonstrated that irradiation induces a drastic shift in microbial community composition, reflected as a distinct clustering pattern observed for irradiated samples. On the other hand, samples of Ubq_Post, Ubq_Pre/Post, and Control groups had a similar clustering pattern, with no clear separation but rather substantial overlap between some samples (Fig. 6). Such pattern indicates that, while irradiation drastically impacts microbial community in rats, Ubq treatments largely restore the community into a structure indistinguishable from non-irradiated rats.

### Taxonomic classification of gut microbiome associated with gamma irradiation and Ubq regimens

**Fig. 7:**
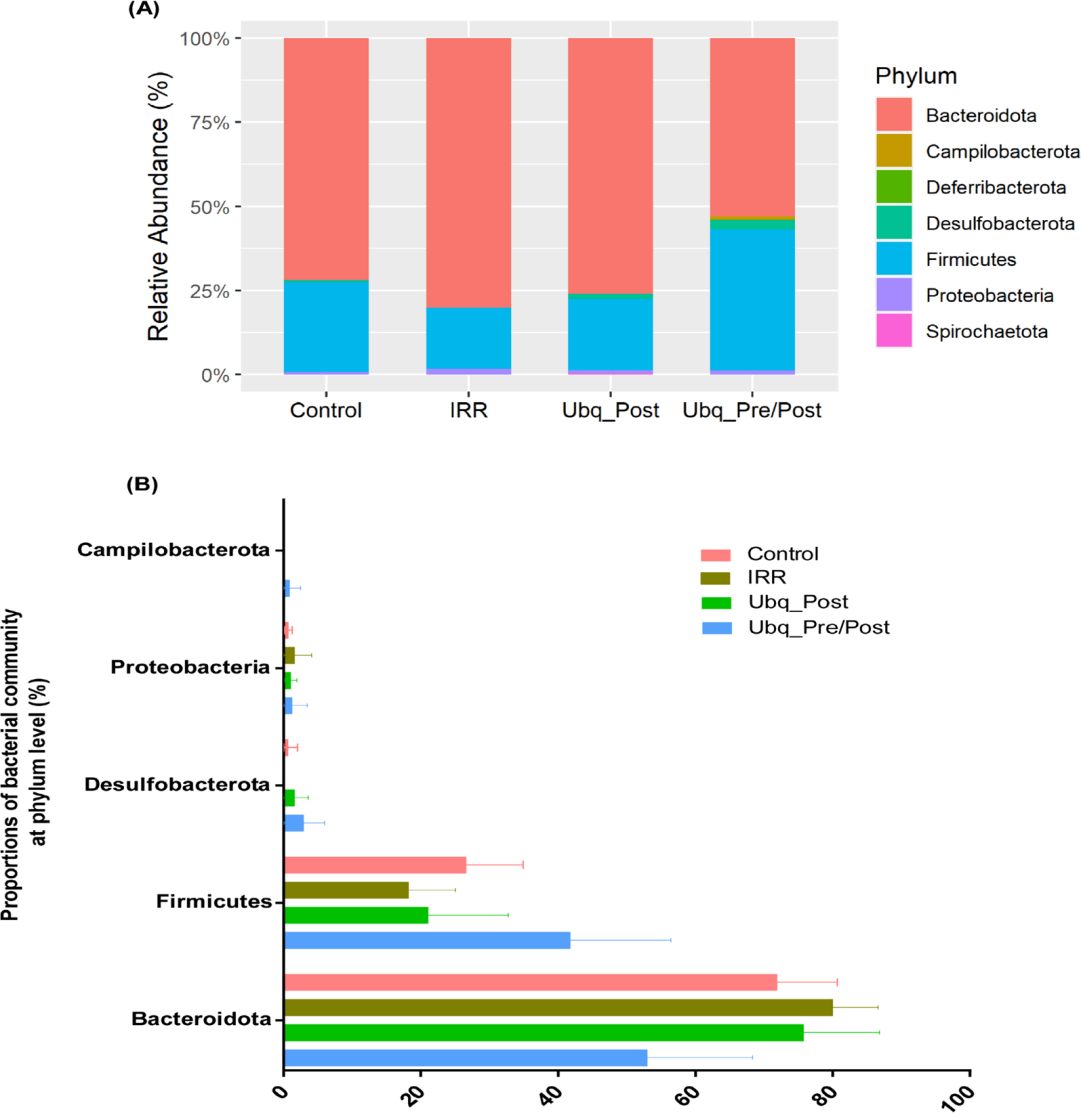
Relative abundance of the major phyla within the bacterial community analyzed. **A** Stacked bar charts illustrating the relative abundance of the detected phyla in the gut microbiome of the animal groups. The X-axis shows the four tested groups, while the Y-axis shows the relative abundance of each phylum detected. **B** Bar charts comparing the relative abundance of bacterial phyla within the different experimental groups. Error bars represent standard deviation of the means

**Fig. 8:**
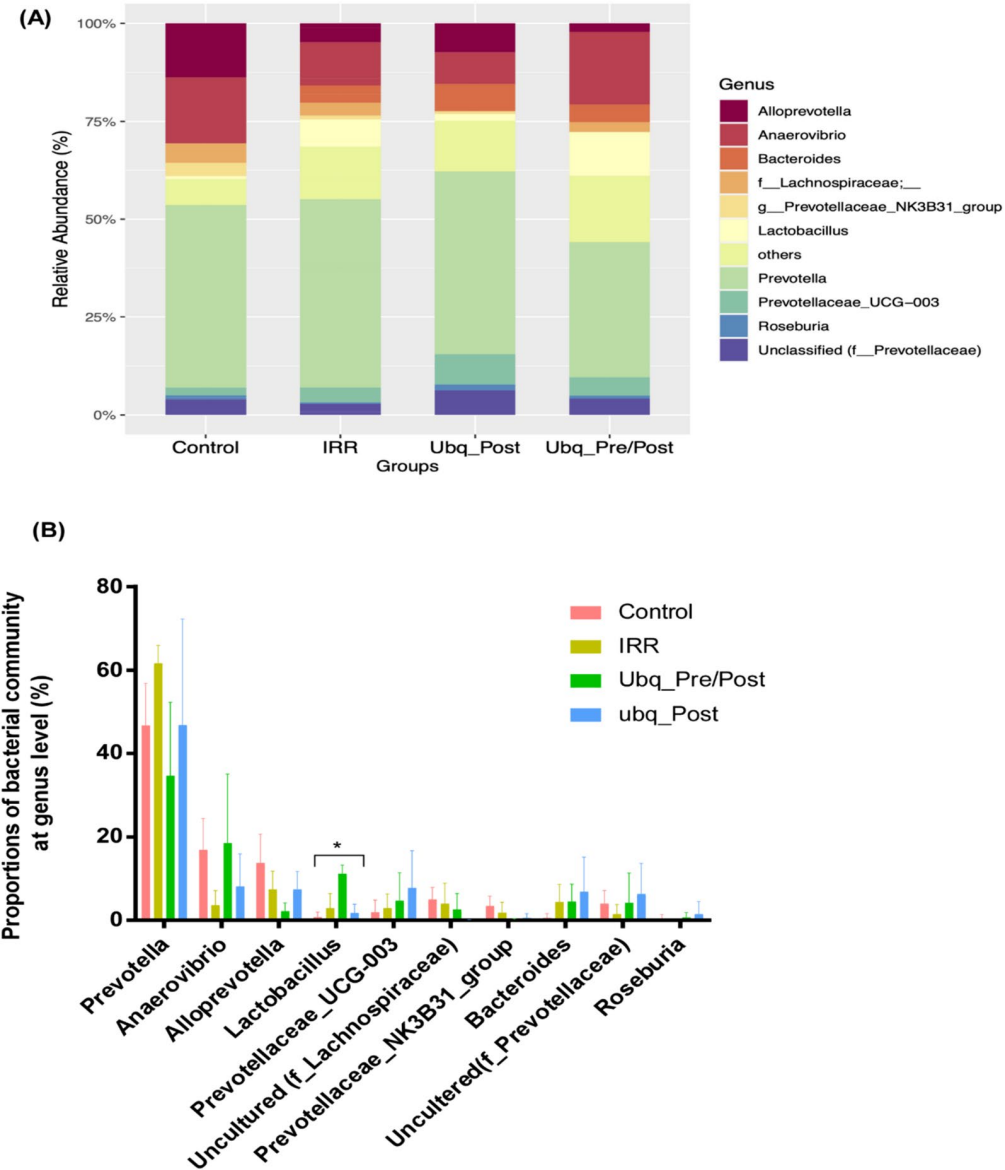
Bacterial community abundance at the genus level. **A** A stacked bar chart illustrates the relative abundance of the detected genera in the gut microbiome of the enrolled groups. The X-axis shows the four tested groups, while the Y-axis shows the relative abundance of each genus detected. **B** Column plots indicating the significance of the top 10 bacterial community at genus level based on Kruskal–Wallis test, followed by post-hoc pairwise Dunn’s testing. Asterisks (*) indicate *p* value ≤ 0.05

The results of taxonomic analysis were to a large extent concordant throughout the four tested groups, as we observed the same pattern of dominance of bacterial taxa (Figs. 7, 8). At the phylum level, seven bacterial phyla were detected (Fig. 7A), with phyla Bacteroidetes and Firmicutes representing more than 90% of sequences encountered in all datasets—a typical signature of mammalian gut microbiota. The mean ratios of Firmicutes to Bacteroidetes were 0.6, 0.27, 0.23 and 0.37 in the Ubq_Pre/Post, Ubq_Post, IRR, and Control groups, respectively.

Moreover, we compared the relative abundance of each phylum with the microbial community (Bacteroidota, Firmicutes, Desulfobacterota, Proteobacteria, Campilobacterota, Spirochaetota, and Deferribacterota) among the four treatment groups, and calculated the significance of these differences which were not statistically significant between the four tested groups (*p* > 0.05) (Fig. 7B).

At the genus level, *Prevotella* was found to have the largest proportion in all samples (Fig. 8A). The combined contribution of four genera (*Prevotella*, *Anaerovibrio*, *Alloprevotella*, and *Lactobacillus*) accounted for more than 70% of the total bacterial community across the four groups. However, statistical analysis only highlighted the *Lactobacillus* genus as significantly different (*p* < 0.05), with a higher relative abundance in the Ubq_Pre/Post group than any of the other three groups (Fig. 8B).

## Discussion

Radiation therapy is crucial for managing cancer and is as part of treatment protocols, as it damages malignant cells' DNA. However, radiation also damages neighboring healthy cells and has detrimental effects on normal tissues, especially those in the gastrointestinal tract, as the intestine represents a large surface area of the human body which cannot be shielded during abdominal or pelvic radiotherapy [1]. Both the intestinal epithelial cells and the gut microbiota hosted on them are affected by the applied radiation [50].

In the current study, male rats were given an acute dose of γ-radiation (7 Gy), which caused structural alterations in the intestinal mucosa seven days after the radiation exposure. These alterations included severe villi damage, notable reductions in villi and crypt lengths, desquamation of epithelial cells, and goblet cell loss. Our results are in agreement with those of Sittipo et al. and Thaiss et al. [24, 51], who demonstrated jejunum histopathological alterations, up to ten days after 6 Gy whole body γ-irradiation. Prior work demonstrated that intestinal damage following exposure to whole body γ-radiation happens in a dose-dependent manner and is characterized by pronounced epithelial cell loss, interstitial submucosa enlargement, and dumping of villi and crypts [9, 52]. However, our pilot histopathological screening showed that the intestinal damage was more pronounced in the jejunum segment of irradiated rats (Supplementary Fig. 1B and Fig. 2B). This was in consistence with two previous studies [53, 54] that reported significant histological damage in the jejunum segment more than the colon segment of irradiated rats exposed to a high dosage of 10 Gy γ radiation. Furthermore, they reported the harmful effects of radiation on the intestinal tissue at least 72 h after exposure to high, but not low (2 Gy) or moderate (5 Gy), radiation doses.

Commonly, most patients suffering from radiation enteritis, especially those receiving pelvic radiotherapy, are also diagnosed with dysbiosis, characterized by reduction in α-diversity and increment in β-diversity, with expansion of Proteobacteria and *Gammaproteobacteria* and depletion of *Bacteroides* [55]. Avoiding gut microbiome dysbiosis after radiation therapy is crucial to achieving optimal oncotherapy, as it could impact protocol therapy and possibly alter radiosensitivity [56]. The gut microbiota was found to play an important role in maintaining the integrity of epithelial cells as well as cell-to-cell connection, which ultimately leads to the restoration of damaged epithelial cells and enterocytes [57]. This led us to the main goal of this study, which is to investigate the possibility of ubiquinol to influence gut microbiome homeostasis, which could be one of ubiquinol protecting mechanisms against radiation enteritis.

Of note, our main objective was to investigate the gut microbiome variations after acute radiation exposure and how it relates to enteropathy. We started by reproducing prior observations in our own animal model, so that we can interpret the microbiome results in context. Prior studies, conducted in different animal models [35, 58] guided us to choose a derivative of CoQ10, because of the latter’s beneficial effect on the gut microbiota. However, we used a different animal strain to test if the results are generalizable or strain specific, and we administered a different form of CoQ10 (Ubiqinol) to the animals, which is a cornerstone of this study. The treatment regimen was slightly different as well. We find that all these factors make the results stronger in context of literature. The accrual of all these findings is important to move from basic to translational research, and subsequently start exploring the possible adaptation of such regimen in humans.

Although we did not directly measure CoQ-10 levels in the rats during the experiment, it is crucial to highlight that the dosage of ubiquinol administered (10 mg/kg/day) was carefully chosen based on recommendations in scientific literature [39, 58, 59]. Additionally, the reason we elected to use ubiquinol, the reduced form of CoQ10, is its superior absorption and bioavailability compared to CoQ-10 [60].

At first, histopathological results showed that treatment of irradiated animals with Ubq, either post- or pre-/post-irradiation, improved the intestinal mucosa, maintained normal villi and crypt length with more pronouncing effect in Ubq_Pre/Post group where inflammatory cells infiltration was undetectable. This was in agreement with a previous study that reported the beneficial administration of reduced CoQ-10 to overcome enteritis in irradiated mice and hypothesized that CoQ-10 accumulated in the intestine, inhibited the damaging effect of ROS on epithelial cells, and maintained length and structure of the villi [35].

The expression of IAP in intestinal tissues was determined in the current study, as it serves as a prognostic factor in gut dysbiosis and enteritis, as IAP deficiency is associated with risks of dysbiosis, intestinal inflammation and permeability [6]. Immunohistochemical staining showed a negative expression of IAP in intestinal tissue, compared to Ubq-treated groups, which showed significant expression of IAP. The anti-inflammatory action of IAP returned mainly to its inhibitory effect on nuclear factor-kappa B (NF-κB), the activator of pro-inflammatory cascade, which is secreted as a result of LPS binding to toll-like receptor (TLR) 4 on immunocytes after destruction of Gram-negative bacteria [61, 62]. Moreover, a previous study [58] supported this finding, as it illustrated the effectiveness of CoQ10 in reducing intestinal NF-κB expression alongside with decreasing inflammatory cytokines, protecting the intestine from further radiation damage [58].

Surging of inflammatory cytokines, in particularlyIL-1β, could result in high intestinal permeability and increased bacterial penetration from the lumen to the lamina propria, which could further induce colitis [63, 64]. In the current study, a significant increase in intestinal IL-1β in irradiated rats supports the luminal barrier self-defense hypothesis, as the disruption of epithelial cells activates the chemokine receptors of mononuclear phagocytic cells to release IL-1β, IL-22 and IL-23 to support the intestinal barrier [29]. Furthermore, the significant reduction of intestinal IL-1β upon treatment of irradiated rats with Ubq agrees with studies which previously reported the anti-inflammatory characteristics of CoQ-10, via different pathways in reducing COX-2, TNF-α and C-reactive protein [58, 65].

In response to the inflammatory state that followed radiation treatment, NO accumulated in intestinal tissue [66]. Our investigation revealed a considerable increase in NO in intestinal tissue seven days after radiation. However, overproduction of NO in response to gamma radiation could be one of the keys to understanding the relationship between radiation-induced enteritis and gut dysbiosis. Under normal conditions, NO is essential to maintain normal epithelial function owing to its potent antimicrobial properties [50]. On the other hand, accumulation of NO may result in different pathogenic bacterial responses according to their sensitivity to it. Some bacteria resist NO-induced damages via the expression of detoxifying enzymes, and some, such as lactobacilli, reduce the level of NO, which was significantly dominant in this study in Ubq_pre/post samples, while others, such as pathogenic *Escherichia coli*, stimulate more production of NO to decrease intestinal barrier permeability and invade host tissue [51, 52].

The significant increases in intestinal NO in our results was accompanied with significant increments in TBARs and caspase-3 levels. These changes suggest the presence of enterocyte inflammation and wall lipid peroxidation with a high incidence of enterocyte apoptosis. Moreover, the abundance of NO, which is among autophagy characteristics, facilitates cell apoptosis and necrosis, and synergistically activates ROS to accumulate in cytosols and mitochondria, leading to extracellular lipid peroxidation and hydrogen peroxide accumulation [58, 67, 68]. On another front, the indirect regulation of gut microbiota composition by antioxidants may be another possible biological mechanism, by which antioxidants influence the diversity and composition of the gut microbiota, ultimately reducing ROS production by activating signaling pathways related to antioxidant enzymes [57]. Although the gut microbiota may enhance gut endothelial apoptotic responses to radiation, some bacteria may instead activate signaling pathways that protect the epithelium from radiation-induced apoptosis, thereby fortifying the intestinal barrier [23]. In the current work, significant reduction of intestinal NO, TBARs and caspase-3 levels were reported in the Ubq_post and Ubq_pre/post groups. Such results can be interpreted in the light of the powerful antioxidant activity of Ubq, which is due to its electron transport capability to produce ATP required for cell energy. This process inhibits membrane lipid peroxidation, thus inhibiting apoptosis [69, 70]. Moreover, CoQ10 regulates the expression of pro-inflammatory cytokines, such as IL-1β and TNF-α, and successfully inhibits the expression of NF-κB, representing a powerful anti-inflammatory treatment [71, 72].

PPAR-γ was further determined in this work as it is considered a sensitive regulator of microbiome homeostasis. Butyrate (a short-chain fatty acid), which is an agonist of PPARs, is produced by the gut microbiome as a fermented product, this process upregulates PPAR-γ according to microbiome activity. However, PPAR-γ’s limited NO production and oxygen bioavailability by directing epithelial cells toward β-oxidation [31]. Our results demonstrate that PPAR-γ was significantly suppressed in irradiated rats, and significantly elevated in intestinal tissue of Ubq_pre/post group. Some studies showed that production of PPAR-γ could be stimulated by different bacterial species, such as *Enterococcous faecalis, Roseburia hominis, Roseburia intestinalis, and Fusobacterium naviforme* [73, 74]. On the other side, some studies showed the inhibitory effect of other species such as *Akkermansia muciniphila* and *Streptococcus salivarius* on PPAR-γ [75, 76]. Moreover, the structural analogy between PPAR-γ and CoQ10 was reported to exhibit a more beneficial regulatory effect on metabolism [62]. Beside the sensitive role of PPAR-γ toward gut microbiome homeostasis, it has an anti-inflammatory activity in pancreatitis cases, which could develop after radiation exposure and carry a high incidence of diabetic risk and intestinal barrier dysfunction [4, 5, 31, 77, 78]. This information is congruent with our finding that Ubq has a beneficial effect on serum insulin levels and intestinal PPAR-γ, which is reflected positively on metabolic rates.

Radiation-induced enteropathy can be seen as a rather complex two-way relationship, as the relation between the gut microbiota and the pathogenesis of radiation-induced gastrointestinal toxicity is believed to be mediated through inflammatory processes, disruption of the epithelial barrier and intestinal permeability, and the release of immune molecules in the intestine. Meanwhile, the resulting dysbiosis has potential immune responses, affecting both localized and systemic levels within the body [79].

It is worth noting that not all microbes are impacted by ionizing radiation in the same way. In addition to cation transport and DNA repair systems, a complex regulatory network that controls bacterial survival and stress adaptation includes post-transcriptional regulators, like small RNAs, which, when properly coordinated, may increase bacterial resistance to ionizing radiation. Dysbiosis may be predicted and prevented with a better understanding of these mechanisms, and identifying which bacteria are more vulnerable to ionizing radiation may help predict and prevent dysbiosis [80].

A variety of outcome measures are commonly used to track variations in diversity, richness, and taxonomic composition between samples. Nevertheless, some consistent findings stand out. Radiation generally reduces the diversity and richness of gut microbiota samples, as well as the relative abundance of specific bacterial taxa. Numerous investigations in the literature reported a decrease in microbial alpha diversity, which subsequent dysbiosis, which is known to increase susceptibility to a range of diseases [80]. Alpha diversity reduction was confirmed in this study, and, although no significant change in beta diversity was observed, clustering of samples in response to treatment with Ubq near the control group might indicate a remodulation of the gut microbiome composition, at least partially, to survive the gamma radiation-induced dysbiotic condition.

Typically, two dominant phyla, the Firmicutes and Bacteroidota make up nearly 90% of the total population of the gut microbiota [57], which was the case in our study. The Firmicutes-to-Bacteroidota (F/B) ratio has been commonly considered as a significant indicator of gut microbiota compositional balance [1]. Yet, in this work, the F/B ratio differences were not statistically significant. While a decrease in this ratio upon radiation exposure (0.23) started to rebound after treatment with Ubq (reaching 0.27), the ratio increased to 0.6 after receiving Ubq for 14 days. Overall, no microbial taxonomic profile could be considered as a signature for irradiation damage or Ubq effects in this study; however, two microbial genera are worth highlighting: (1) *Prevotella* was the dominant genus in this study, albeit without a conclusive overabundance in a particular group. Genus *Prevotella* is one of the most abundant gut genera in rats as well as humans, and has been associated with numerous health conditions, but cannot be resolved by 16S studies alone [81]. Its abundance here is consistent with literature and confirmatory of a typical basal gut microbiome profile in rats. (2) *Lactobacillus* was the single bacterial genus to be statistically significant overabundance in one group: the Ubq_Pre/Post group. Such observation needs further investigation in future studies.

## Conclusion

Our study highlights the importance of considering multiple factors when studying radiation-induced enteropathy in experimental rats. In particular, considering the changes that occur to the gut microbiota is important to understand the side effects of abdominal and pelvic radiotherapy. The study indicated a consistent trend suggesting that Ubq treatment improves the general intestinal health, including the diversity and balance of the gut microbiota. This improvement did not significantly differ, in most instances, when Ubq was used before- and after- or only after-radiation exposure. With larger population studies, better identification of confounding variables, and further investigation of the inflammatory response affecting the gut microbiome, a more comprehensive picture of the mutual irradiation-Ubq-microbiota interactions will be obtained. To the best of our knowledge at this stage, Ubq can be successfully and safely used either as radioprotective or radiomitigator to alleviate the inflammatory response caused by radiotherapy.

### Supplementary Information

Supplementary Material 1.
