# Supplementary material for: Gastroprotective and microbiome-modulating effects of ubiquinol in rats with radiation-induced enteropathy
Source: Anim Microbiome. 2024 Jul 19;6:40. doi: 10.1186/s42523-024-00320-9 (PMC11264694; doi:10.1186/s42523-024-00320-9)
Supplement: Supplementary file 1 — Supplementary Material 1. [file 42523_2024_320_MOESM1_ESM.docx]

**Gastroprotective and microbiome-modulating effects of ubiquinol in rats with radiation-induced enteropathy**

**Walaa A. Eraqi ^1*^, Walaa A. El-Sabbagh ^2^, Ramy K. Aziz ^1,3^, Mostafa S. Elshahed ^4^, Noha H. Youssef ^4^, Nora M. Elkenawy ^2*^**

* Authors to whom correspondence should be addressed:

**Walaa Ahmed Eraqi**

Department of Microbiology and Immunology, Faculty of Pharmacy, Cairo University, Cairo, 11562, Egypt

[walaa.eraqi@pharma.cu.edu.eg](mailto:walaa.eraqi@pharma.cu.edu.eg)

Mobile number +20 127 973 7917

**Nora Mohamed Elkenawy**

Drug Radiation Research Department, National Center of Radiation and Research Technology (NCRRT), Egyptian Atomic Energy Authority (EAEA)

Cairo, 11787, Egypt

[nelkenawy@gmail.com](mailto:nelkenawy@gmail.com)

Mobile number +201223560870

| **[A] Control** | **[B] IRR (7^th^ day)** | **[C] IRR (14^th^ day)** |
| --- | --- | --- |
| 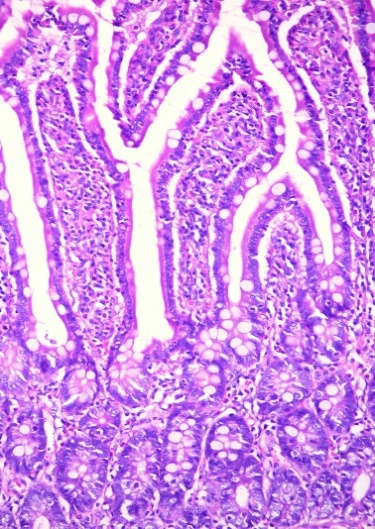 | 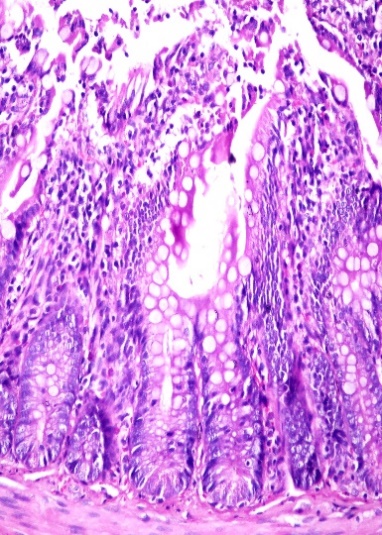 | 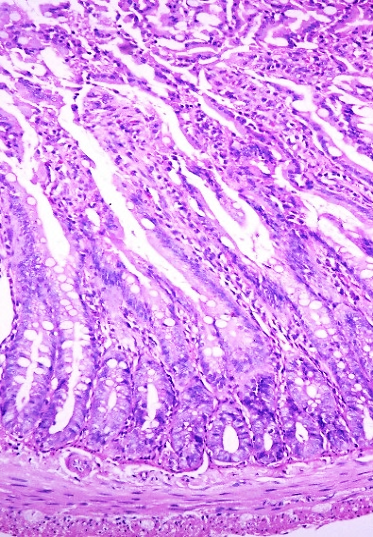 |

**Figure S1:** Photomicrograph of a cross section in the jejunum showing [A] normal histological structure of intestinal mucosa in **Control** group, [B] severe damage of intestinal villi with loss of goblet cells after 7 days from exposure to γ-radiation (7 Gy), [C] moderate desquamation of epithelial lining with loss of goblet cells after 14 days from exposure to γ-radiation (7 Gy), (H&EX200).

| **[A] Control** | **[B] IRR** | **[C] Ubq_Post** | **[D] Ubq_Pre/Post** |
| --- | --- | --- | --- |
| 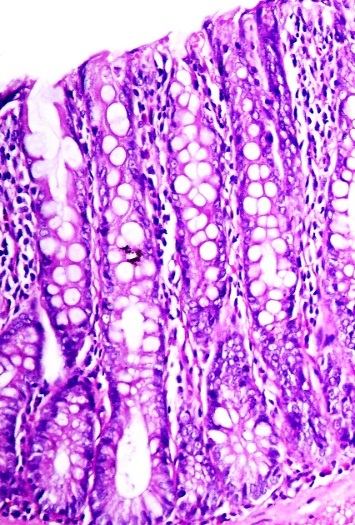 | 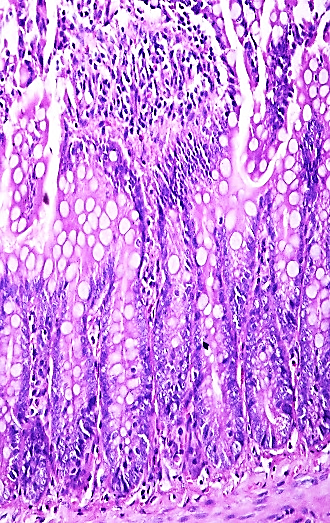 | 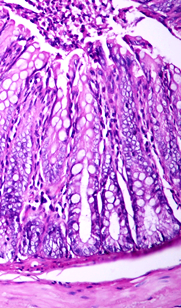 | 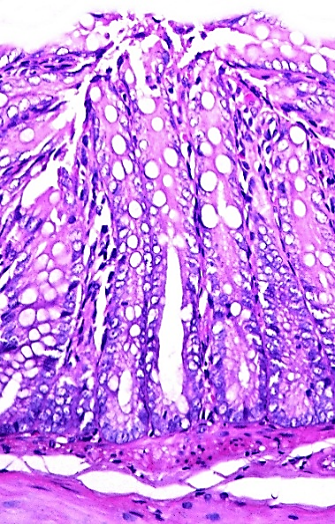 |

**Figure S2:** Photomicrograph of colon tissue section 7 days post irradiation, was showing [A] normal histological structure of colon mucosa in **control** group, [B] loss of villus architecture and mononucleolar cells infiltration in **IRR** group, [C] mild damage of colon villi with kept villus architecture in **Ubq_Post** group, [D] Intact epithelial lining of colon crypt in **Ubq_Pre/Post** group (H&EX200).


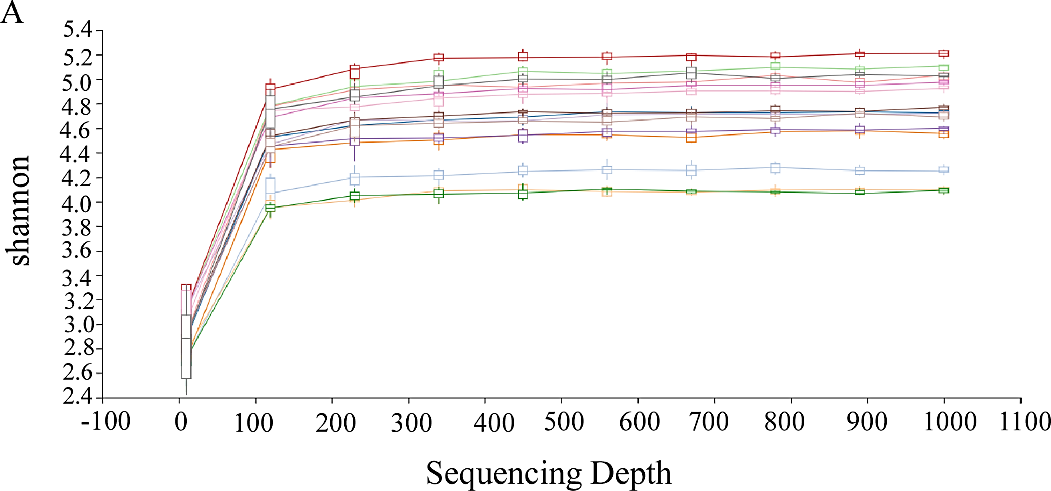


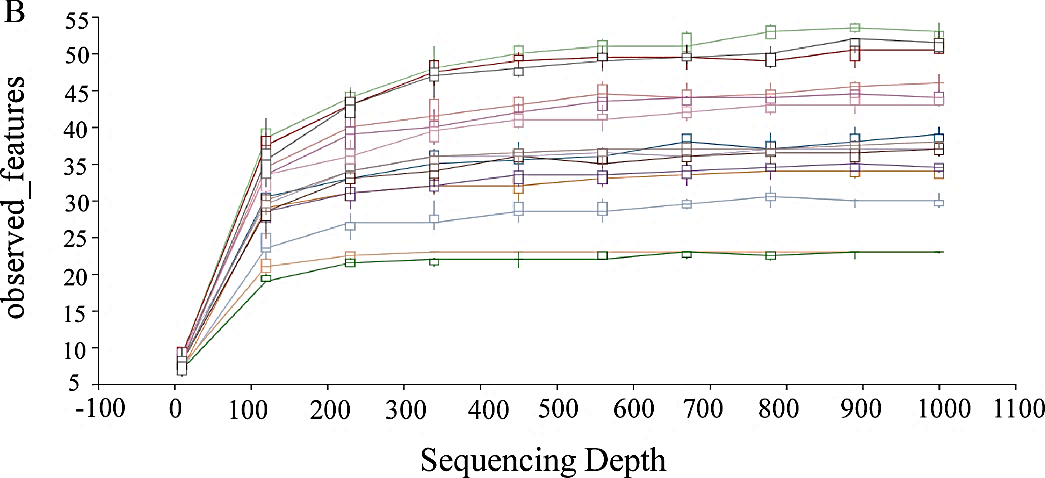


**Figure S3: Individual rarefaction curves for gut microbiome sampled from the four experimental groups. (A) shows the Shannon and (B) shows the number of observed features**
